# The influence of empowered work environments on the psychological experiences of nursing assistants during COVID-19: a qualitative study

**DOI:** 10.1186/s12912-020-00489-9

**Published:** 2020-10-16

**Authors:** Jasmine L. Travers, Krista Schroeder, Allison A. Norful, Sainfer Aliyu

**Affiliations:** 1 Rory Meyers College of Nursing, New York University, 433 1st Avenue, New York, NY 10010 USA; 2 Temple University College of Public Health, 3307 North Broad Street, Philadelphia, PA 19140 USA; 3 Columbia University School of Nursing, 560 West 168th St., New York, NY 10032 USA; 4 MedStar Washington Hospital Center, 110 Irving St NW., Washington, DC, 20010 USA

**Keywords:** Patient care technician, Frontline workers, Acute care, Direct care, Coronavirus, Empowerment

## Abstract

**Background:**

Nursing Assistants (NA) who feel empowered tend to perform their duties better, have higher morale and job satisfaction, and are less likely to leave their jobs. Organizational empowerment practices in hospitals likely shape the psychological experiences of empowerment among these personnel; however, little is known about this relationship.

**Objective:**

We used qualitative inquiry to explore the relationship between organizational empowerment structural components and feelings of psychological empowerment among hospital frontline workers during a public health emergency.

**Methods:**

Kanter’s Theory of Structural Empowerment and Spreitzer’s Psychological Empowerment in the Workplace Framework were applied to identify the conceptual influences of organizational practices on psychological experiences of empowerment. In-depth interviews were conducted with a convenience sample of NAs, caring for hospitalized COVID-19 patients. Directed content analysis was performed to generate a data matrix consisting of the psychological experiences of meaning, competence, self-determination, and impact embedded under the organizational structural components of information, resources, support, and opportunity.

**Results:**

Thirteen NAs (mean age = 42 years, 92% female) completed interviews. *Information*, or lack thereof, provided to the NAs influenced feelings of fear, preparation, and autonomy. *Resources* (e.g., protocols, equipment, and person-power) made it easier to cope with overwhelming emotions, affected the NAs’ abilities to do their jobs, and when limited, drove NAs to take on new roles. NAs noted that *support* was mostly provided by nurses and made the NAs feel appreciated, desiring to contribute more. While NAs felt they could consult leadership when needed, several felt leadership showed little appreciation for their roles and contributions. Similar to support, the *opportunity* to take care of COVID-19 patients yielded a diverse array of emotions, exposed advances and gaps in NA preparation, and challenged NAs to autonomously develop new care practices and processes.

**Conclusion:**

Management and empowerment of healthcare workers are critical to hospital performance and success. We found many ways in which the NAs’ psychological experiences of empowerment were shaped by the healthcare system’s empowerment-related structural conditions during a public health emergency. To further develop an empowered and committed critical workforce, hospitals must acknowledge the organizational practice influence on the psychological experiences of empowerment among NAs.

## What is already known about the topic?

- Nursing assistants are important to patient care delivery, yet are undervalued, underutilized, and often poorly treated.
- Empowerment of nursing assistants has been suggested as an approach to improving morale and job satisfaction among nursing assistants thereby leading to decreases in turnover and better patient outcomes.

## What this paper adds**?**

- This paper demonstrates how organizational structures and practices influence nursing assistants psychological experiences of empowerment.
- This paper outlines how the contributions of nursing assistants may be maximized.

## Introduction

The U.S. is in the midst of a rapidly evolving pandemic crisis with 7,475,262 COVID-19 cases to date [1]. At the start of this crisis, the American healthcare system proved to be ill-prepared for the influx of COVID-19 patients who required treatment. Hospitals were faced with overwhelming demands and scarce resources to provide care for a population with which they had no experience [2, 3]. Those who spend the most time in patient care (i.e., nursing assistants [NA]) are critical to the quality of care and decision-making efforts in the face of uncertainty [4]. NAs have the potential to make significant contributions to patient care, yet they are underutilized, understudied, and commonly disregarded [5].

NAs comprise an essential part of the patient care team. The patient support that NAs provide enhances the quality and comfort of a patient’s stay at a healthcare facility [6]. For example, they not only provide physical care for patients, but also emotional and spiritual support. They are the eyes and ears of the clinical staff and can readily alert them of changes or declines in a patient’s condition.

Despite the role NAs play in patient care, they experience abuse, disrespect, challenging physical labor, exclusion from important conversations, limited opportunities for advancement, and underpay [5]. These experiences have led to an unhappy and overworked direct care workforce, increased turnover, and inefficient processes. Over the past two decades, there has been a push to elevate the role of NAs and underscore their value so they ultimately feel empowered [7]. The empowerment of NAs might lead to greater participation in planning, decision-making, and interdisciplinary teams, all activities being required of NAs across healthcare settings [8]. Importantly, NAs who feel empowered tend to perform their duties better, have higher morale and job satisfaction rates, and are less likely to consider leaving their jobs [9–11]. The benefits of an empowered NA workforce are especially important during a crisis in which quickly changing organizational practices related to empowerment are likely to shape the psychological experiences of empowerment; however, little is known about this relationship.

Based on Kanter’s Theory of Structural Empowerment, organizational leadership must create empowered environments that optimize work output by ensuring that employees have access to important information, resources, and support that enable them to perform their jobs well and provide opportunities for employee growth and development [12]. We sought to understand the changing roles, contributions, and experiences of NAs during the COVID-19 pandemic at a large urban academic medical center by exploring the relationship between organizational empowerment practices and the psychological experiences of empowerment among this personnel.

## Methods

### Study design

This qualitative inquiry is a sub-analysis of a larger study focused on understanding public health crisis preparedness and the response of healthcare workers within a hospital system (Aliyu S, Norful A, Schroeder K, Oldlum M, Travers JL: The powder keg: lessons learned about hospital preparedness during the COVID-19 pandemic, submitted).

### Theoretical framework

**Fig. 1:**
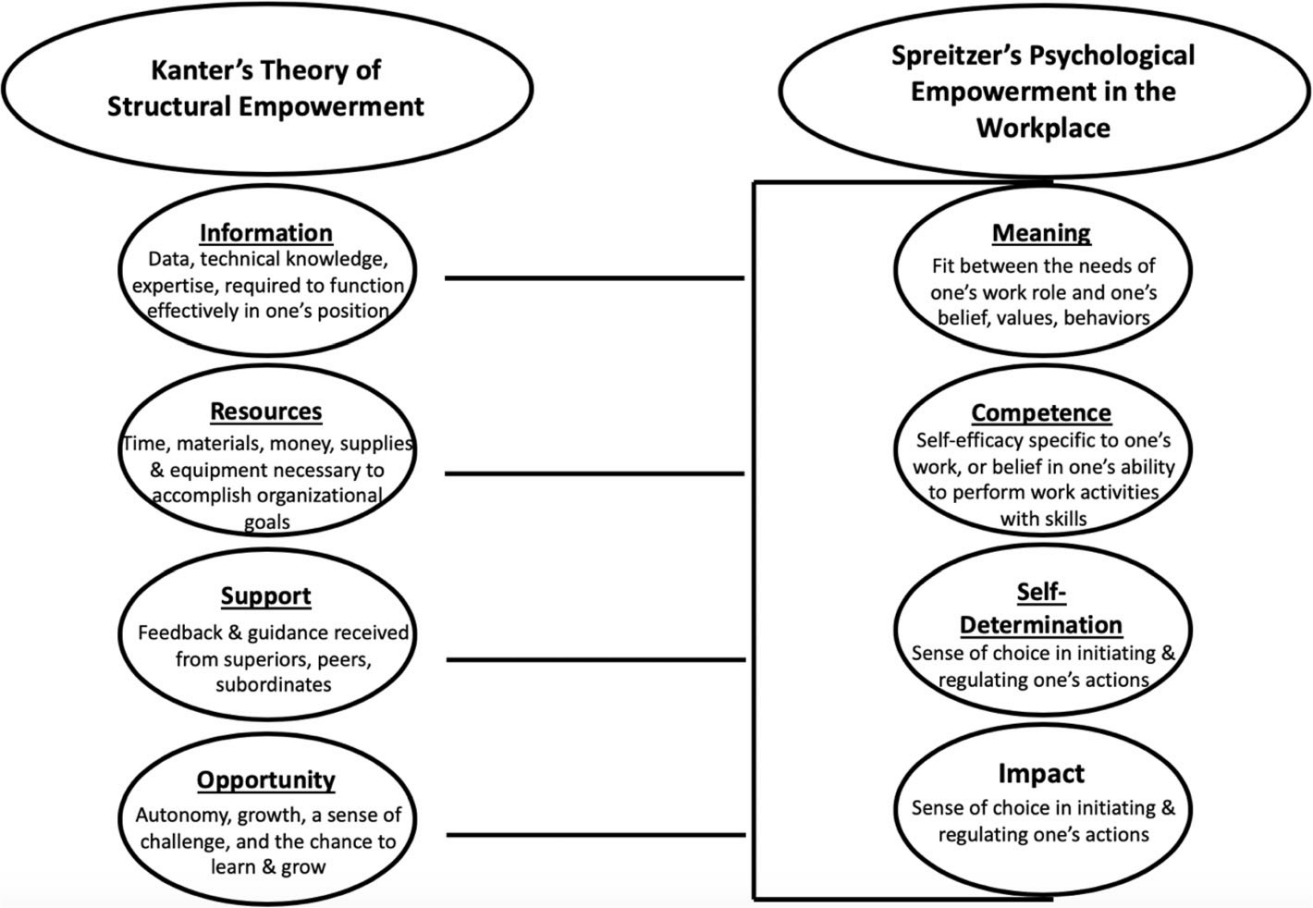
Kanter’s Theory of Structural Empowerment and Spreitzer’s Psychological Empowerment in the Workplace Framework Applied to the Present Study

Kanter’s Theory of Structural Empowerment and Spreitzer’s Psychological Empowerment in the Workplace Framework were applied to identify the conceptual influences of organizational practices on psychological experiences of empowerment among NAs (Fig. 1) [12, 13]. The organizational practices that enable an employee to perform well as described in Kanter’s Theory include information, resources, support, and opportunity [12]. The psychological experiences of empowerment described in Spreitzer’s Framework include meaning, competence, self-determination, and impact [13]. *Meaning* is the alignment between one’s work role and their beliefs, values, and standards. *Competence* is one’s belief that they can carry out their work duties. *Self-determination* is one’s sense of autonomy or control in initiating action. *Impact* is one’s belief that they can make a difference in the organizational processes.

### Sample and recruitment

NAs caring for COVID-19 patients in a large urban academic hospital center were the population of interest. Recruitment of NAs was conducted by the study Principal Investigator (PI), who was a healthcare administrator of the hospital at the time of the study. The PI visited hospital units with COVID-19 patients and approached potentially eligible NAs requesting their participation in the study. NAs were given information about the study and assessed for their eligibility to participate: 1) ≥18 years old; 2) English-speaking; 3) able to complete a one-time, in-person, one-on-one interview; and 4) caring for or cared for a patient diagnosed with COVID-19.

### Data collection/study procedures

In-depth, one-on-one, semi-structured interviews were conducted by the PhD-prepared nurse female PI (SA), among a convenience sample of NAs who consented to participate in the study during March and April 2020. Interviews took place in a private location at the hospital at a time convenient for the NAs, with no one else present. A 21 question open-ended interview guide informed by Donabedian’s Quality Framework for Evaluation of Healthcare Delivery directed interview sessions (see Supplemental File A) [14]. This interview guide was developed for the larger study. Interviews lasted approximately 25 min. Data collection continued until no new information emerged from the interviews (i.e., data saturation). Field notes were taken after the interviews. Qualitative interviews were audio-recorded, transcribed verbatim, and analyzed using NVivo 12. The Institutional Review Board at MedStar Health Research Institute approved the study.

### Data analysis

Directed content analysis was performed by two coders to organize the data into a matrix consisting of the psychological experiences of meaning, competence, self-determination, and impact embedded under the organizational structural components of information, resources, support, and opportunity. Coding was initially driven by these theoretical dimensions, but also progressed to emergent themes. A primary coder initiated coding and discussed preliminary findings during weekly research meetings. Team members provided input on coding and category assignment decisions. This process took place iteratively until consensus was achieved for all coding and category assignments. Once the codebook was finalized, the remainder of the coding continued. Twenty percent of the transcripts were double-coded by a second researcher on the research team. The team had prior experience conducting qualitative interviews and analysis [15–18].

Approaches to ensure rigor included credibility, dependability, confirmability, and transferability. Member checking was used to achieve credibility. During the interviews, the PI repeated information back to the participant to ensure a shared understanding of the data. For dependability, our team maintained a substantive audit trail of all study activities, including the data collection process. Reflexivity was important to ensure the confirmability of our data. Weekly research meetings allowed researchers to discuss their interpretations of the findings and confirm the originations of data interpretations. For transferability, we sampled a wide range of NAs across units to ensure that the selected participants represented the variety of views of NAs across units [19].

## Results

Thirteen NAs (average age = 42 ± 11.90 years, 92% female) completed qualitative interviews at which time saturation was reached. NAs averaged nine years working in the profession and the majority of them (69%) were assigned to medical-surgical units. All provided care to COVID-19 patients regardless of the unit that they were assigned to. Additional descriptives of the NAs can be found in Table 1. The NAs discussed the many ways in which their psychological experiences of empowerment related to meaning, competence, self-determination and impact were influenced by the hospital’s organizational empowerment practices related to information, resources, support, and opportunity. The psychological experience of impact did not emerge under the information and support constructs. Exemplar quotes can be found in Supplemental File B.

**Table 1:** Demographic Characteristics of the Sample of Nursing Assistants (NAs)

	Total (*n* = 13) n (%) or mean ± std
Individual Characteristics	
Race/Ethnicity	
White	1 (7.7)
Black	10 (76.9)
Hispanic	2 (15.4)
Age, *years*	42 ± 11.90
Gender (Female)	12 (92.3)
Certification	
NA Certificate	10 (76.9)
NA (ED Tech)/EMT Certificate	3 (23.1)
Years in Profession	9.4 ± 8.86
Years in Current Role	5.9 ± 5.69
Unit/Department	24.0 ± 4.3
Med Surg	3 (23.1)
ICU	3 (23.1)
Med Surg/ICU	1 (7.7)
Med Surg/Float Pool	3 (23.1)
ED	3 (23.1)

*STD* Standard Deviation, *NA* Nursing Assistant, *ED* Emergency Department, *EMT* Emergency Medical Technician, *ICU* Intensive Care Unit

### Information

Information related to training, preparation, and practice in addition to knowledge influenced how NAs described meaning, competence, and self-determination.

#### Meaning

Information provided by leadership directly impacted how NAs created meaning around their work efforts. While NAs processed information delivered to them, such as knowledge and training about the care of COVID-19 patients, they found that they had to process this in the context of how it affected them. NAs discussed fear, frustration, and the need to pray. Others felt that “this is the job” (NA 4007) and “this is what I signed up for,” (NA 4009) highlighting the importance of empathizing with the patient. One NA stated, “I was concerned for myself, but I replaced the concern for myself with more compassion for the patients” (NA 4002).

#### Competence

Information delivery also influenced how competent NAs felt in doing their jobs. Information was typically shared via huddles and emails, consisting of knowledge or training related to COVID-19 patient care. Several NAs discussed feeling unprepared to do their jobs because of the lack of adequate job knowledge and training. For example, a NA who received informational material without any direct contact stated, “Well, we looked at it a little on the computer, a video, and that was it. And they brought a book in here.. . I don’t think it’s very helpful” (NA 4013). NAs wanted to be shown rather than told. “The manager told us what to wear, but nobody showed us. It would be good to have some practice” (NA 4007). In particular, NAs suggested having more practice with personal protective equipment (PPE). “I know we should probably know this, but it’s a lot right now and we are panicking and want to make sure we do it right to protect ourselves” (NA 4005). To compensate for the lack of training, NAs discussed learning from each other.

Preparation included being mentally prepared to take on a new responsibility. A NA described having “no mental preparedness at all, because when I was confronted with the situation like, hey, we have a COVID-19 confirmed on our unit and it’s gonna be your patient, I was literally shaking. I was sweating. I still feel like I have the same feeling because it was kind of hard handling mentally and I’m gonna worry I’m not prepared enough to take care of a person. I don’t know how the patient is gonna be. Will the patient ask me things that I don’t know? Do we have the tools or enough preparation to take care of this person?” (NA 4001).

#### Self-determination

When NAs received adequate information, they felt confident. “I feel pretty much confident now because after they tell us, ‘Hey, this is what you’re gonna wear.’ They tell us, ‘This is what you’re gonna do” (NA 4001). This information sharing could have occurred in one-on-one meetings with direct supervisors or educators informing the NAs on how to wear their PPE. Having someone “to guide you and talk” (NA 4009) was most beneficial to NAs.

The lack of information or inadequate information forced NAs to feel self-determined to acquire the information on their own such as knowledge and training that they were not receiving or challenge information that they did not agree with. When acquiring information on their own, NAs discussed seeking training whether it was via videos or other mechanisms in which to educate themselves. A NA shared, “We have not been given training on the unit. But I have shown myself a video teaching myself how to properly use PPE, to put it on and take it off” (NA 4006).

When given information that NAs were conflicted about, they felt determined to speak up against it. For example, a NA described, “We sit outside but the door is slanted, so we can see the patient from outside, because even if it is a negative pressure room, it still isn’t safe for us to sit in this small room, and they were telling me I had to sit in that room. I said, ‘No.’ Even my boss said, ‘Oh, it’s safe.’ I said, ‘Safe? This is a new disease. I don’t think that was fair to us” (NA 4011).

### Resources

Having resources such as time, materials, money, supplies, equipment, and staff necessary to accomplish organizational goals additionally affected the meaning, competence, self-determination, and impact that NAs experienced.

#### Meaning

Access to proper resources such as PPE was important for alleviating NAs’ fears. Particularly, NAs recognized the importance of PPE for their protection and that the lack thereof predisposed them to serious risks. “If you do not know and if you do not find the PPEs that you know that you need to wear, go to your manager or the trainers and ask for it. And, I guess, you will be safe” (NA 4009).

NAs noted that it was more than just having this resource at your disposal and knowing what to put on “because literally, you feel like you are putting yourself at risk in that type of situation” (NA 4001). Additionally, NAs noted that the lack of help took a toll on them. For example, a NA noted that “the COVID patients are heavy, so an extra NA would be nice” (NA 4005).

During the early phase of the pandemic, NAs shared how unhappy they were about limited testing and available PPE. “I’m just thinking about the projection that 60% of healthcare workers will get it. And then I think about my baby. And it’s not that we’re not being tested is what I’m upset about, but I ask where’s the plan? I don’t hear a potential plan that we’re even thinking about for workers” (NA 4005). NAs did not feel they were given the same consideration as other hospital personnel.

#### Competence

NAs had difficulty accessing protocols or guidelines that would enable them to adequately care for COVID-19 patients. They also discussed wanting a better understanding of how to access materials. This affected their abilities to do their jobs. “Well, we can’t do our job because we have to make sure we’re properly gowned up and everything before we can still work with the patients. Say if they code, we won’t have the proper materials. So, I think we should get a little extra stock of the PPE” (NA 4003).

#### Self-determination

NAs took the initiative to leverage their resources to enhance their learning and keep themselves updated. For example, a NA noted, “I mean, there are resources, like [the hospital intranet], and we are looking them up at the hospital, what the protocols are and all that we need are in [the hospital intranet]. I’m checking [the hospital intranet] too to keep myself updated” (NA 4006). If staffing was limited across personnel type, NAs discussed taking on additional roles. For example, one NA discussed housekeeping not being the greatest at pulling linen as a result of limited staff, “so usually I pull trash to keep the unit tidy and the rooms clean. I have a whole system going on” (NA 4003). While not part of their formal responsibilities, the NAs would use the computer to understand their patients. One NA noted, “I always look in the computer in the mornings to see who’s positive and who’s negative, so I prepare myself. And I just gear up and get everything I need to go in there” (NA 4013).

#### Impact

NAs appreciated the impact they had on patients, but recommended greater stewardship of staffing resources. A NA proposed, “Better staffing, actually thinking the schedule through—like the way they staff and who they staff with—like if you staff one particular part of the ER with nurses who are immunocompromised who can’t take COVID patients.. .. You know you have some of the nurses that are stronger—who can handle more stuff. You have some of the weaker nurses. So, it kind of falls back on us, the techs, because you have nurses that really can’t multitask with very sick patients. And then you’ll have us and they say, ‘Okay, well can you go in this room and get this line,’ even though this one is a young PUI (person under investigation)” (NA 4008).

### Support

Support included feedback and guidance received from superiors, peers, family and friends along with mental support.

#### Meaning

Support influenced how NAs felt about their roles and this support made them feel like a part of a team and appreciated. This support could come from anyone such as registered nurses, peers, leadership, or family. “They are very supportive. Everybody, today especially, I am very thankful to all the nurses. They are full of confidence. Everybody’s helping each other. We are more likely to be friendly and supportive, now. We used to, but now it’s more. We are like a team. I feel wonderful about that. Even though you have a fear, people give you positive energy, and you feel okay having to take care of a COVID-19 patient” (NA 4006). The nurses were described as helping more than usual. “I’m gonna overall say the nurses help a lot more then they typically would…It’s just about multitasking and working as a team to try to keep everybody safe. We communicate more” (NA 4002). Another NA said, “It was quite exciting. I hope this experience will continue on throughout the duration of healthcare” (NA 4002). “I feel good when we work as a team. It makes it easier and you’re not tired as much as when you’re working alone. We can better help each other” (NA 4011).

NAs felt like they were seen. “I’m thanked more. Even though I feel like I don’t deserve it because I’m just doing my job, I feel like we’re a little bit more of a family with the nurses” (NA 4010). Coworkers checked in more frequently as a result of the pandemic with questions like, “Hey, are you okay? Do you need something? Do you want me to do this for you? How does the patient feel?” (NA 4001). They supported each other so that they did not feel overwhelmed.

NAs received prayers or mental support from their family as well as from nurses on the unit, noting that spiritual support helped. “There is a nurse on the floor, I love her. She was like, ‘It’s okay. You’re gonna get through it. This too will pass.’. .. Her spirit is just awesome for the unit, for the patients. So, when I think about spiritual support, I really go to her” (NA 4005). Others created a prayer team on the unit. “We can just come up there and pray before we start our shift” (NA 4002).

Lack of support from leadership left NAs feeling abandoned and upset. One NA described, “I worry for my son; I was crying because my son has a respiratory disease and he was intubated at 13 months. I told that to my director, but she didn’t care, at least that’s how I felt. She told me to take the patients and had me using the regular masks. I was very upset about that” (NA 4005). Even just talking to NAs more or expressing appreciation would have been enough for NAs. NAs discussed being provided some safety precautions for COVID-19 but still wanted more like temperature checks as they left for the day (NA 4008). They wanted to know that people cared for them. “I could care less about the free food, about the money, about the hazard pay if I’m working in an environment where I know my managers and my peers and my coworkers care for one another or at least for me as a human being.. .. You care most about the numbers, about how things look on the outside versus how we actually feel” (NA 4008). It was also not being respectful of and supporting the social needs of NAs. “I am also pulling linens and cleaning a lot of rooms. One new change is that we are to come in and leave in street clothes. But that’s hard. I take the shuttle bus and it leaves on time. We are supposed to change 15 minutes before the shift ends to catch the bus, but there have been times when I change and the nurses call me to go into a patient’s room. That’s not fair, because they see me already in my clothes, so I have to get back in my uniform and go in the room” (NA 4013).

#### Competence

NAs had several questions as they drew on the support of leadership, nurses, and other disciplines to feel competent in doing their jobs. “I spoke with one of the supervisors. As I was going into the patient’s room, every time that I was going in there, I was coming out with a thousand questions. I was like, okay, let’s talk to my supervisor and see if this is or not okay to do. How do I approach this situation with the patient? At this point, I’m not gonna tell you that I feel 100 percent sure or safe going into those rooms but I’m gonna tell you that I’m not feeling scared the same way as I felt at the beginning” (NA 4001). In order to get the full information required to do their jobs, NAs looked to the nurses. “My main support, I look to my nurses. They have a little bit more communication with the doctor. They have a little bit more communication with respiratory. They have a little bit more different avenues of information coming in. More than just the general information. So, you don’t always take the word for the gospel, but you try to make sense out of nonsense. So, you just take little things here and there to make sure that you’re doing what you’re supposed to do” (NA 4005).

Being short-staffed was discussed as an impediment to carrying out one’s job but having the support of nurses made a big difference. NAs talked about feeling confident in the support from leadership and physicians. In the absence of obtainable leadership support, there seemed to be someone available like a resource nurse or staff nurse. NAs specifically noted that training and mentorship would help. “But it just would be nice if there was the training and somebody to guide you and talk along the way, but without the training, we learn from each other, because we are so close. As a family department we are so close” (NA 4009).

#### Self-determination

NAs felt well-supported by the nurses thus making them feel comfortable approaching nurses to assist them with their duties. “Like today, I talked to several of them. They don’t mind doing it because they are already in the room, so instead of me getting dressed, they are okay with that. But if they need a bed bath or some other additional thing, then I will go in and help them” (NA 4006). This support allowed NAs to take on and learn additional roles. “Housekeeping gets the trash and the nurse strips the room, but housekeeping is barely coming so I help the nurse sometimes. They show me how to strip the room and clean the cords so I do a lot for them” (NA 4006).

In addition, communication increased where there was minimal to no communication previously. “That’s actually something that’s been showing up a lot and is very present. Because let’s say I’ll communicate to the nurse, ‘Hey, I’m going to the patient room. Do you want me to bring anything to the patient? Do you want me to tell something to the patient? Is there something that you want me to do that I can do or vice versa?’ The nurse tells me, ‘Hey, I’m going to the patient room. Do you wanna do something? Do you want me to ask the patient this? Do you want me to get water? Do you want me to check if the patient needs something or things like that?’ It’s something that has changed in the communication between us. We have very good communication most of the time, and handling a COVID-19 patient has improved it in a good way” (NA 4001). NAs made it a point to look out for others and be supportive. “It’s heightened by the need to take strict precautions, and we do look out for one another. They make sure that we have the equipment on correctly. And lately, I’ve been seeing the RNs by our side going in with us. EKG techs have gone in, phlebotomy have gone in. And I do communicate with them, just to make sure each of us are safe when we go in to see the patient” (NA 4012).

### Opportunity

The opportunity to take care of COVID-19 patients and be a part of something new to NAs challenged them, allowing them to grow and take on new autonomous roles.

#### Meaning

At the height of the COVID-19 pandemic, the meaning of the NA role was altered. Meaning reflected the NAs’ different emotions regarding this new opportunity and how it changed how they viewed their jobs and roles.

NAs were affected emotionally as they took on this new opportunity, and similarly to other personnel, they were concerned about risk. “I care for my son. He’s at home. I don’t wanna take it home. I try to take any precaution that I can while I’m here, but I don’t wanna take it home” (NA 4006). These concerns affected initial reactions and encounters of how the NAs approached patients. A NA discussed their initial reaction as “fear, of course. I’m scared still, but I gotta do what I gotta do” (NA 4006). A lot of this fear was present leading up to the first patient, but a few patients in, the fears subsided. In general, NAs did not want to risk bringing the virus home to family.

Despite being alerted about caring for COVID-19 patients, approaching something new still left NAs feeling vulnerable, anxious, stressed, sleepless, and overwhelmed. Some NAs did not want to accept COVID-19 patients while others recognized this change as part of their job, despite deciding to distance from family and friends. NAs, like other healthcare personnel, needed to make family adjustments with living situations.

NAs experienced difficult cases that were emotionally challenging. However, they recognized the importance of taking care of themselves. “You want to make sure that you walk the fine line between taking care of yourself, but also taking care of the patient. You’re here to do a job, definitely, but you’re also a person. And you have feelings and emotions, and you’re entitled to those.” (NA 4005).

NAs’ roles during the COVID-19 pandemic were ambiguous for some. One NA shared, “I’m not gonna say there’s not much for me to do, but like I said, there’s no clear role on what I should do as a tech when a COVID patient comes in other than to get access or get a line or get the blood” (NA 4008).

*Prayer* and *faith* were a big part of coming to terms with this change. “Just pray as much as you can because I feel like that’s all you can do because there’s people that I know that have it and they’ve been in full PPE” (NA 4008). Another NA shared, “At first, I had concerns and yes, a little fear. But I’m a Christian and I put my faith in God and I just know that he’s protecting us here. And I pray for all of us that are working with the patients” (NA 4012). NAs discussed praying at the start of the shift as the only thing that they did differently when taking on this new responsibility (NA 4002). “It’s not up to me. It’s God, a higher power, whatever you believe in, whatever I believe in, it’s not up to me” (NA 4010).

NAs in the emergency department (ED) seemed to be more expectant of the uncertainty ahead. One NA had “no concerns because you prepare for this already. So, we are in the frontline, so you are prepared to have whatever comes into the door. And you should be able to handle it, no panicking” (NA 4009). These NAs in the ED already had the mindset that you care for whatever presents itself. “Well, because the ER cannot close the door to people. Sometimes people keep coming and coming, so I know I have to take care of them” (NA 4009).

#### Competence

Working with COVID-19 patients changed how NAs felt about their ability to perform their jobs. Many expressed that they were doing the best that they could and intended to take care of their patients “just like any other patient” (NA 4001) and that they were “prepared to have whatever comes into the door” (NA 4009). Although this duty was new, knowing that they were following the guidelines, no matter how the situation turned out, provided comfort to the NAs.

While being able to do their job was challenging when it came to caring for a new patient population, the ability to care for these patients improved over time. One NA explained, “For the first time, we didn’t understand a lot.. .. We didn’t know exactly what to have or what to do. We kind of learned from the first patient that we had, so we got a little better with it after that first batch” (NA 4003). Despite increasing competence, NAs still wanted to confirm they were doing things right. “But you just want to make sure you walk that fine line because you want to do what you’re supposed to do. You want to make sure that you’re taking care of the patient. That you’re not treating the patient any type of way. And you also want to make sure that you’re doing what you’re supposed to do to keep COVID with that patient” (NA 4005).

#### Self-determination

Because this situation was so dynamic, NAs could redefine and fully step into current, new, and advancing roles. For example, NAs began to assume roles that consisted of teaching and correcting others. “We can teach everybody how to properly don PPE. And we just saw someone just walking without PPE, so someone walking out with PPE, dirty PPE, so you need to be more aware and ready to teach respiratory, NA, nurses, everybody, how to use it” (NA 4006). Another NA noted that adherence to PPE rules differed between disciplines. “No one knows. Someone was walking with a surgical mask and with a glove, and I yelled at her. And she was a physician, but I was watching out for everyone’s safety. The patient was a PUI, but we didn’t know he tested negative. We just found out when she told us the result after I yelled at her. I didn’t mean to yell at her but she could expose us” (NA 4006).

In these cases, NAs spoke up. “I am very vocal. So, if I do not understand something or I see something, I try to educate other people that do not have the right PPEs with them. And some of the people get it like, ‘Oh, I really appreciate it.’ Some of the people, they do not like it because they think you hurt them feeling like, ‘Oh, I mean, I know what I am doing.’ But, no, you are not wearing the proper PPEs, you should be wearing this” (NA 4009).

NAs shared tips and tricks for improving patient care, such as clustering care, and highlighted the importance of communication. Clustering care for the NAs consisted of ensuring that they had everything they needed when entering rooms. It was important to limit visits to the room and work as a team. “We are trying not to go into the rooms too much. I have 14 patients, and I will be the one who is going to all the rooms.. .. I will tell the nurse to do it while in the room to save the PPEs for my unit, for the hospital, for everybody else” (NA 4006). When clustering care, communication between nurses and NAs was critical. A NA described her communication with a nurse. “There is a lot more communication when you have a nurse that has a COVID patient” (NA 4005).

NAs stepped up to help when others were not performing duties and took initiative to create opportunities that would make everyone’s job easier. “I made up a bag that we could put in each patient’s room, so we don’t have to go back and forth, in and out of the room.. .. That bag has everything to intubate the patient to put the pole in, put in the A-line, dialysis kit, central line kits, everything in that one bag” (NA 4003).

NAs were more careful and strategic during this time, recognizing the importance of the needs of COVID-19 patients. “Whereas a positive COVID patient, you want to make sure that you are staying on top of everything, no matter what it is. Whether it’s their breathing. Whether it’s their mobility. Whether it is their sugar. Do they have a fever today, a fever yesterday? You just want to make sure that you’re monitoring their progress. So, it just feels more intense and urgent” (NA 4005).

NAs also listened more to coworkers from other disciplines. “I find myself listening to respiratory more. What they have to say. How they are going about it. The ideas that they have, whether they are talking to me, themselves, or the nurse. You just kinda try to listen to what’s going on….I find myself really paying attention to respiratory” (NA 4005).

NAs made themselves available to other personnel caring for patients. “And everybody’s jumping, not very prepared, because you know, when somebody calls, you act quickly. And then I always try to help them, like to wear proper PPE, and I always try to stand by the door and give them what they need like masks and gown” (NA 4007).

NAs took ownership of patients and their statuses. In addition to looking up patient information on their own, NAs kept a closer-than-normal eye on their patients. “Going into the room, we are looking at the patient—if the patient is a higher risk than normal, we take special precautions and tell the nurse that so-and-so is not doing so well. We watch respirations because COVID patients have trouble with that and we tell the nurses if the number is, say, over 24 or 30, whatever they tell us. We assist all the nurses on the floor because we have to use our eyes too. We make sure staff wear the right PPE to go in the rooms, we look out for that too, it protects everyone” (NA 4004).

NAs were determined to stick up for and protect themselves, all while finding a voice. When wearing a mask on a pre-COVID-19 unit, a doctor questioned why one NA was wearing a mask. “I said, ‘Because a lot of patients are coughing and they don’t cover their mouth, and I have to protect myself from them as well them from me, you know, if I’m coughing,’ so she said it didn’t look appropriate, it gives the wrong impression…Two weeks later, who did I see with a mask on?” (NA 4013).

#### Impact

In this new situation, NAs felt they could make an impact on the patient as well as the organizational practices through recommendations. Regarding a patient, a NA noted, “I wasn’t here when she got worse, but I wish I could’ve been there for her again” (NA 4005), knowing that they had provided comfort that the patient needed.

Recommendations for managing COVID-19 patients included housing patients in the same location. “I know that this is kinda ridiculous to say that all COVOD-19 patients be in one area instead of having some here and some there. You don’t know sometimes what you’re getting into. You’re in this patient’s room, and this patient is clear. By the time you step off this one, you’re onto another patient who is positive” (NA 4004).

## Discussion

In this qualitative study, we sought to understand how NAs’ psychological experiences of empowerment were influenced by the organization’s structural levels of empowerment. To our knowledge, this is the first study to look at these constructs among a sample of NAs employed in the hospital. We summarize how the structural components of information, resources, support, and opportunity influenced NAs’ experiences of meaning, competence, self-determination, and impact during an emergent response to the COVID-19 pandemic. Our findings are important for informing long-standing efforts aimed at enhancing the role of the NA. This enhanced role might consist of NAs working to their full potential, increased involvement among NAs in patient care activities and the interdisciplinary teams, and collaborative relationships between nurses and NAs.

*Information*, or lack thereof, provided to the NAs influenced feelings of fear, preparation, and autonomy and seemed to affect NAs’ feelings of competence. These feelings were similar to those expressed in other novel and unprecedented crises such as the 1980’s AIDS epidemic [20]. In-service training, a form of information sharing, has been found to be associated with reductions in workers’ reported stress, perceived risks, and negative attitudes, with subsequent improvements in knowledge and satisfaction with the quality of care provided during times of crises [21]. However, as technology and a focus on efficiency becomes more profound in our society, in-services delivery methods have changed. In-services might now come in the form of pamphlets or via online platforms [22]. Yet, NAs in this study did not just want to be handed or provided information; they wanted to be shown and provided this information in the form of face-to-face contact. These findings highlight the need to retain in-person trainings and ensure that NAs understand the information that they are receiving.

*Resources* such as protocols, equipment, and staff, made it easier for NAs to cope with overwhelming emotions, facilitated the NAs’ ability to do their jobs, and when limited, drove NAs to take on new roles. Taking on new roles, such as those that usually fell under housekeeping and environmental services, meant additional responsibilities for NAs. Moreover, scarcity of resources was a common complaint across hospital systems during this COVID-19 pandemic [3, 23]. Important attention to resource stewardship, especially before becoming constrained, expanding the workforce, and staff protection is necessary [24]. Critically, many frontline direct care workers (e.g., NAs) did not receive the same protections and supportive resources that registered nurses and physicians received during the COVID-19 crisis such as housing, discounted transportation, and meals/groceries [2]. American media coverage often focused on the role of nurses and physicians, which may have led to a decreased appreciation for the role of team members such as NAs. This may have also impacted how supportive resources were allocated.

*Support* was mostly discussed by the NAs as being provided by nurses and made the NAs feel appreciated with the desire to contribute more. Support was deeply rooted in the evolved exchanges between NAs and nurses. This relationship consisted of listening, communicating, and collaborating in ways that have been advocated for by many for decades [25, 26]. While NAs expressed they could ask leadership questions when needed, several NAs shared that they felt leadership did too little to show that they valued their role and contributions. These findings highlight opportunities that are important for hospitals to capitalize on as well as areas to improve in. Particularly the opportunity for leadership to recognize and honor NAs through mechanisms beyond, pay, training, and incentives. Additionally, NAs, like many other healthcare personnel, were experiencing emotional hardships that tended to be overlooked when compared to other healthcare personnel. Recognizing that NAs have similar responses and experiences as nurses when it comes to burnout and stress is important when considering the need for mental health support [27, 28]. NAs discussed prayer and prayer teams as significant support mechanisms that helped them to cope.

Similar to support, the *opportunity* to take care of COVID-19 patients yielded a diverse array of emotions, exposed advances and gaps in the preparation of NAs, and challenged NAs to autonomously develop new practices and processes of care. This development of new practices and processes of care allowed NAs to innovate and contribute greatly to the care of COVID-19 patients between the ideas of clustering care and developing kits. They used this opportunity to make an impact on patient care and also provided recommendations for change. NAs spoke up when they felt the situation warranted their voice and made it a point to understand the care needs of their patients by initiating activities such as looking up their patients’ charts or inserting themselves into conversations with important team members such as respiratory therapists. These findings affirm the need to include NAs in discussions around optimizing patient care delivery (Travers JL, Caceres BA, Zaidi H, Vlahov D, Stone PW: Improving Nursing Home Quality through Certified Nursing Assistants’ Full Participation in Resident Interdisciplinary Care Planning and Teaming, submitted).

### Implications

COVID-19 undoubtedly revealed weaknesses, challenges, and shortfalls in our healthcare system, but also opportunities, growth, and successes. Very importantly, we see how to maximize the contributions of NAs and meet their intrinsic needs. To capitalize on the strides made, our findings have important implications for policy and practice change. It is important that attention is placed on how information related to knowledge and training is disseminated across all disciplines and that hospital leadership ensure that all parties, including NAs, receive that information in a clear and detailed manner. Attention to the mental health and social needs of NAs nationwide is also needed to recognize that like nurses and physicians, these frontline workers are at risk for experiencing burnout and stress along with other threats to mental health. Considering the current pandemic, more support staff may be needed to protect NAs from burnout and relieve understaffed nurses of routine healthcare tasks. It is additionally important to provide support across the board. Incentives, increased pay, ladder opportunities have been recommended for enhancing the role of the NA, but it is important that we do not forget that NAs want to be seen, heard, and valued from the top down as discussed in this study. There is also a need to focus on creating a supportive and caring environment for NAs where they feel empowered to communicate and collaborate with other personnel. To further develop an empowered and committed critical workforce, hospitals must acknowledge the organizational practice influence on the psychological experiences of empowerment among direct care healthcare workers.

### Limitations

Because the interviews were conducted by a healthcare administrator employed by the hospital, the NAs could have felt a power dynamic that restricted their voice. However, the healthcare administrator reflected the same racial background of the majority of the NAs who participated and many were excited to share their experiences. Further, she functions as the Director of Nursing Research and has no supervisory role to the participants. Additionally, our data comes from a convenience sample in one hospital which limits our ability to generalize the study findings across the larger population of hospitals. Although not generalizable, this work is significant and timely as it highlights long-standing concerns surrounding the NA’s role and emotional well-being. To our know knowledge these issues have never been examined in this manner. Hence, our findings could provide guidance on a larger scale for empowering NA’s and improving care delivery.

## Conclusion

Management and empowerment of NAs are critical to hospital performance and success as empowered NAs have been shown to improve their job satisfaction, performance, morale, and retention. We found many ways in which the NAs’ psychological experiences of empowerment were shaped by the healthcare system’s empowerment-related structural conditions during a public health emergency. This included the need to: 1. Convey information to NA’s to alleviate fears, enable them to be prepared, and allow for them to be autonomous, 2. Ensure access to resources among NAs to facilitate their ability to do their jobs which is critical as they too are responsible for a significant portion of the patient’s care, 3. Provide support to NAs which could be seen as the institution creating a team environment inclusive of NAs, checking in on NAs, and recognizing NAs for their contributions as NAs want to feel valued, and 4. Allow NAs to innovate and contribute to the decision-making process when new opportunities present themselves in the workplace as these healthcare personnel have important insights as well. Attention to the role that prayer and spiritual support plays in the psychological experiences of NAs is additionally important.

## Supplementary information

**Additional file 1: Supplemental File A.** Interview Guide, This file provides questions and probes that were used to facilitate data collection during the interviews with the nursing assistants.

**Additional file 2; Supplemental File B.** Exemplar Quotes, This file provides exemplar quotes representing the emerging themes.

## Data Availability

The datasets used and/or analysed during the current study are available from the corresponding author on reasonable request.

## Abbreviations

ED: Emergency Department
EMT: Emergency Medical Technician
ICU: Intensive Care Unit
NA: Nursing Assistant
PPE: Personal Protection Equipment
PUI: Person Under Investigation
SD: Standard Deviation
