# Supplementary material for: The influence of empowered work environments on the psychological experiences of nursing assistants during COVID-19: a qualitative study
Source: BMC Nurs. 2020 Oct 16;19:98. doi: 10.1186/s12912-020-00489-9 (PMC7561701; doi:10.1186/s12912-020-00489-9)
Supplement: Supplementary file 1 — Additional file 1: Supplemental File A. Interview Guide, This file provides questions and probes that were used to facilitate data collection during the interviews with the nursing assistants. [file 12912_2020_489_MOESM1_ESM.docx]

Supplemental File B. Interview Guide: Questions w/ Probes

1. Tell me about your home and family.
2. Tell me about your work.
3. Tell me the story of when you realized that you would need to care for COVID-19 patients?
   1. Did you hear anything from your leaders?
4. Tell me about your preparation and training to care for patients diagnosed with covid-19.
   1. Did you feel you were prepared with enough knowledge and training?
   2. Are senior personnel available to answer questions that you might have?
   3. Do you have access to protocols?
5. Tell me about the resources you have access to that are supposed to protect you from acquiring COVID-19 while in the hospital?
6. Describe your process when caring for a COVID-19 patients. What do you do?
7. How has your role changed since the COVID-19 crisis?
8. Tell me a little about the hospital influences and actions pertaining to COVID-19 patients. What members of the healthcare team do you interact with? What were their roles?
9. Describe changes to your team in preparation for COVID-19.
10. How would you describe the support from organizational leaders?
11. At the time of your first COVID-19 patient assignment, what was your initial reaction?
    1. What were your fears or concerns?
12. How have you been treated by friends, family, peers and the media in general?
13. What type of psychological support have you received, if any?
14. How’s your mental health?
15. How would you rate your physical health before and after the start of this pandemic?
16. How are you sleeping?
17. How would you rate your feelings of burnout, if any at this point?
18. What environmental or organizational characteristics increased or decreased your feelings of burnout?
19. Can you give me an example of anything traumatic you have seen or experienced pertaining to the COVID-19 pandemic while at work?
20. What advice stemming from your own experience do you have for others that may be caring for COVID-19 patients?
21. Is there anything else you’d like to share?
