# Supplementary material for: The influence of empowered work environments on the psychological experiences of nursing assistants during COVID-19: a qualitative study
Source: BMC Nurs. 2020 Oct 16;19:98. doi: 10.1186/s12912-020-00489-9 (PMC7561701; doi:10.1186/s12912-020-00489-9)
Supplement: Supplementary file 2 — Additional file 2; Supplemental File B. Exemplar Quotes, This file provides exemplar quotes representing the emerging themes. [file 12912_2020_489_MOESM2_ESM.docx]

| Supplemental Appendix. Exemplar Quotes | | | | |
| --- | --- | --- | --- | --- |
|  | **Meaning-** fit between the needs of one’s work role and one’s belief, values, behaviors. *Prayer, family, emotions- fear, stress, overwhelmed, makes it easier, the potential of bringing COVID to family, don’t know one’s role, this is your job, this is my job* | **Competence-** refers to self-efficacy specific to one’s work, or belief in one’s ability to perform work activities with skills. *Feeling prepared, confident, characteristics mentioned that would help the work, protect oneself, doing the best we can, questions* | **Self-determination-** a sense of choice in initiating & regulating one’s actions (making decisions about, work, effort, pace). *Evolving role. I have to do my job. One’s process when initiating an activity* | **Impact**- the degree to which one can influence strategic, administrative, or operating outcomes at work. *Providing strategic recommendations* |
| **Information-** data, technical knowledge, expertise, required to function effectively in one’s position. *Training,* *preparation, practice* | “Then my director told us we will have these patients. I don’t like that we will become a COVID floor because I don’t want to have these patients all the time. I have a son and I’m scared.” (NA 4005) | “This educator came. She informed us about the PPE, especially the N95 to wear when the patient is positive, make sure you have those in perspective and put on properly, how to fix your gown and how to take it off and on. They teach us how to do it. So, that makes me feel more comfortable doing it.” (NA 4004) | “But if I work in Faster Care – like before things have changed, before the COVID clinic – most of the people ask for the Faster Care and you know I was swabbing them. I was doing everything and I wasn’t taught how to properly swab them and you know and the procedures. I just knew I had to gown up and then it’s like, “Oh, it’s like a flu test.” (NA 4008) | N/A |
| **Resources-** time, materials, money, supplies, & equipment necessary to accomplish organizational goals. *Guidelines, protocols, person-power* | “Probably send a little extra help to NAs. One for one side of the unit and one for the other side would help. Any help will do, even if it’s not from a NA, any help. It’s going from room to room changing, putting clean linen, gowning up, taking off, going to the next room. So, I mean, it’s a lot.” (NA 4003) | “I guess I have to make do. The most important thing we have is the masks and the gown and the gloves.”  (NA 4001) | “Well, I always leave the sign on the door. Because at first, we had to sign, initial that we entered the room with the time, and the time we came out.” (NA 4013) | “I stated earlier like I still have a fear. I wish we had more full coverage– like the gown we have, it's does not fully cover your back, so I wish we have one of the yellow ones that covers your whole entire uniform.”  (NA 4006) |
| **Support-** feedback & guidance received from superiors, peers, subordinates. *Includes family and friends, treatment by others, mental support, NAs feel that they can ask questions. NAs supporting the nurse. Working with others. Having a system in place where people are working together* | “Yes, for instance, they’re going to get medication. We also need to take vitals. They would say you guys don’t need to come in the room to do the vitals because I’m already there. So, I’m gonna give medication and also take the vitals. That will eliminate both of us from being in the room at the same time. The nurses appreciate us more and work with us more since COVID.” (NA 4004) | “Coming from a nursing home, you have one nurse that has 16 patients. And then more times than not you’re short-staffed. So, to come to a hospital where my nurse has four patients and if I need her, she’s there. She’s available to me. The support from day one, I’ve raved about it. It’s such a difference.”  (NA 4005) | “That one time, was like, not COVID patient, but she’s PUI, and she called. And everybody’s jumping, not very prepared, because you know, when somebody calls, you act quickly. And then I was like try to help them, like to wear proper PPE, and I always try to stand by the door and give them what they need like masks and gown.” (NA 4007) | N/A |
| **Opportunity-** autonomy, growth, a sense of challenge, and the chance to learn & grow. Having the opportunity to take care of COVID-19 patients, process of caring for a COVID-19 patient | “My husband would say just make sure you are doing the right thing. I’m used to wearing my uniform home. Now because of this, I change. I wear my street clothes to go home. I don’t wear my uniform anymore. I don’t bring it into the house because I’m the only person that works in healthcare. So, I don’t wanna take anything home.  ‘that is what we signed up for, so’ (NA 4004) | “When it comes to dealing with the patients, I feel confident. I feel equipped. I feel confident in what we’re doing here at the hospital. I feel confident in the staff that we have. And the support that we have in my leaders, the physicians, and things like that.”  (NA 4005) | “Well, the first time the patient wasn’t intubated, sedated or none of that, so we had, they had to get a line put in. I made up a bag that we could put in each patient’s room, so we don’t have to go back and forth, in and out of the room.” (NA 4003) | “I know that this is kinda probably ridiculous to say that all COVID-19 patients be in one area instead of having some here and some there. You don’t know sometimes what you’re getting into. You’re in this patient’s room, and this patient is clear. By the time you step off this one, you’re onto another patient who is positive. So, it’s vice versa.” (NA 4004) |

Note. N/A is used where no themes emerged. NA= nursing assistant, PUI=person under investigation. Red indicates where definition was enhanced based on data.
